# Syntaxin interacts with arachidonic acid to prevent diabetes mellitus

**DOI:** 10.1186/s12944-022-01681-3

**Published:** 2022-08-18

**Authors:** Undurti N. Das

**Affiliations:** 1 UND Life Sciences, 2221 NW 5th St, Battle Ground, WA 98604 USA; 2 Department of Biotechnology, Indian Institute of Technology, IITH Road, Sangareddy, Kandi, Telangana 502285 India

**Keywords:** Syntaxin, Arachidonic acid, Lipoxin A4, Pancreatic β cells, Insulin, Diabetes mellitus, Docosahexaenoic acid, Resolvins, Protectins, Maresins, Hypothalamus, Inflammation, Exocytosis, Membrane fluidity

## Abstract

Syntaxin regulates pancreatic β cell mass and participates in insulin secretion by regulating insulin exocytosis. In addition, syntaxin 4 reduces IFNγ and TNF-α signaling via NF-ĸB in islet β-cells that facilitates plasma glucose sensing and appropriate insulin secretion. Arachidonic acid (AA) has potent anti-inflammatory actions and prevents the cytotoxic actions of alloxan and streptozotocin (STZ) against pancreatic β cells and thus, prevents the development of type 1 diabetes mellitus (induced by alloxan and STZ) and by virtue of its anti-inflammatory actions protects against the development of type 2 diabetes mellitus (DM) induced by STZ in experimental animals that are models of type 1 and type 2 DM in humans. AA has been shown to interact with syntaxin and thus, potentiate exocytosis. AA enhances cell membrane fluidity, increases the expression of GLUT and insulin receptors, and brings about its anti-inflammatory actions at least in part by enhancing the formation of its metabolite lipoxin A4 (LXA4). Prostaglandin E2 (PGE2), the pro-inflammatory metabolite of AA, activates ventromedial hypothalamus (VMH) neurons of the hypothalamus and inhibits insulin secretion leading to reduced glucose tolerance and decreases insulin sensitivity in the skeletal muscle and liver. This adverse action of PGE2 on insulin release and action can be attributed to its (PGE2) pro-inflammatory action and inhibitory action on vagal tone (vagus nerve and its principal neurotransmitter acetylcholine has potent anti-inflammatory actions). High fat diet fed animals have hypothalamic inflammation due to chronic elevation of PGE2. Patients with type 2 DM show low plasma concentrations of AA and LXA4 and elevated levels of PGE2. Administration of AA enhances LXA4 formation without altering or reducing PGE2 levels and thus, tilts the balance more towards anti-inflammatory events. These results suggest that administration of AA is useful in the prevention and management of DM by enhancing the action of syntaxin, increasing cell membrane fluidity, and reducing VMH inflammation. Docosahexaenoic acid (DHA) has actions like AA: it increases cell membrane fluidity; has anti-inflammatory actions by enhancing the formation of its anti-inflammatory metabolites resolvins, protectins and maresins; interacts with syntaxin and enhance exocytosis in general and of insulin. But the DHA content of cell membrane is lower compared to AA and its content in brain is significant. Hence, it is likely DHA is important in neurotransmitters secretion and regulating hypothalamic inflammation. It is likely that a combination of AA and DHA can prevent DM.

## Introduction

Secretion of adequate amounts of insulin is needed to regulate plasma glucose levels and thus, maintain energy homeostasis. For this purpose, β-cells need to sense and respond adequately to changes in blood glucose levels to prevent hypoglycemia or hyperglycemia. To produce these important actions of insulin: (i) the generation of metabolic signalling molecules; (ii) the regulation of β-cell membrane potential; (iii) insulin granule dynamics and exocytosis and (iv) expression of adequate number of GLUT receptors is needed. This implies that optimal β cell response to plasma glucose is dependent on the pancreatic β cell membrane integrity, dynamics, and composition. This is since, crosstalk between the genome and the cell membrane is essential as all the stimuli impinging on the cell membrane need to be conveyed to genome and vice versa [1]. This implies that cell membrane structure and its integrity and consequently its functions are crucial to receive and send signals to the external environment and in the present instance to the synthesis and secretion of insulin.

### Arachidonic acid (AA) and lipoxin A4 (LXA4) have anti-diabetic actions

**Fig. 1:**
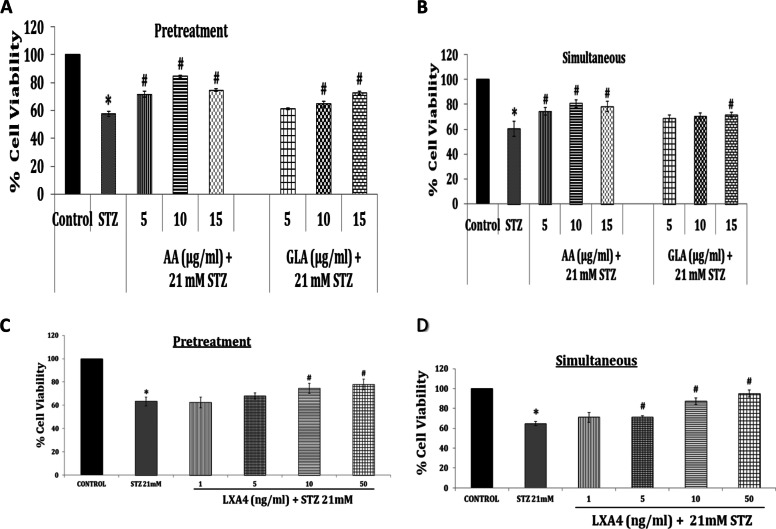
Effect of GLA, AA and LXA4 on STZ-induced cytotoxicity to RIN (rat insulinoma) cells in vitro. Studies were done using both pre-treatment (where RIN cells were pre-treated with GLA, AA and LXA4) and then exposed to STZ; and simultaneous treatment schedule (where RIN cells were exposed to both STZ and lipids at the same time). AA is effective in both pre-treatment and simultaneous schedules whereas GLA is more effective in pre-treatment schedule (but is still less effective than AA). It is seen from these results that AA is more potent than GLA in preventing apoptosis induced by STZ. LXA4 is almost equally effective in preventing STZ-induced apoptosis of RIN cells in both pre-treatment and simultaneous treatment schedules. This data is taken from reference 5. STZ = Streptozotocin; GLA = Gamma-linolenic acid; AA = Arachidonic acid); LXA4 = Lipoxin A4

It is well known that polyunsaturated fatty acids (PUFAs) when incorporated into the cell membrane increase its fluidity (of the cell membranes) [2, 3]. In contrast to this, cholesterol and saturated fatty acids increase the rigidity of the cell membrane [1, 2]. Increased cell membrane fluidity enhances the number of insulin receptors and their affinity to insulin and thus, augments insulin action and reduces the development of insulin resistance and development of type 2 DM [2, 3]. It was reported that *fat-1* mice (that can convert n-6 to n-3 PUFAs) are protected against STZ–induced type 1 DM. This beneficial action has been attributed to suppression in the production of pro-inflammatory cytokines (IL-1β, TNF-α), inhibition in the expression of NF-kB, increase in the expression of GLUT-2 receptors, and enhanced generation of lipoxin A4 (LXA4), a metabolite of AA [4]. Our studies that revealed that AA and LXA4 prevent alloxan and STZ-induced type 1 and type 2 DM in Wistar rats are in support of these findings ([5] and see Fig. 1). Similar beta cell protective action of AA and LXA4 was seen against the cytotoxic action of benzo(a)pyrene, doxorubicin, and alloxan [6] suggesting that AA and LXA4 possess broad spectrum of cytoprotective action. In addition, AA prevented the palmitic acid (PA) mediated cytotoxicity to clonal HIT-T15 pancreatic β cells [7] and restored impaired insulin expression and secretion. These results are interesting in the light of the fact that saturated fatty acids increase insulin resistance and contribute to the pathobiology of type 2 DM, whereas unsaturated fatty acids prevent the development of DM [1–3]. This is supported by the observation that PA induced cell death can be prevented by AA but not by monounsaturated fatty acids including palmitoleic acid (POA) and oleic acid (OA). It is noteworthy that PA induced cell death by producing mitochondrial membrane potential loss that is effectively blocked by AA and thus, prevented apoptosis. Furthermore, AA significantly rescued PA-impaired glucose uptake and -signal transduction of Akt in response to insulin [7–9]. In an invitro study, it was reported that AA generated competently cellular droplets at low concentration within the cytosol of myotubes (C2C12 myotubes) compared with POA and OA. This led to the suggestion that incorporation of harmful PA into inert triglyceride (TG) may be the reason for the protective effects of AA against PA-induced lipotoxicity. This was supported by the observation that in the presence of AA, PA was rapidly channeled into AA-driven TG droplets. Thus, AA diverts PA into inert TG that accounts for its cytoprotective action [7–9]. In addition, AA has potent anti-inflammatory actions [5, 6]. Our studies (in vitro using rat pancreatic insulinoma cells-RIN cells; and animal studies performed in Wistar rats that were induced to develop type 1 and type 2 DM by STZ injection) revealed that administration of AA enhances the production of LXA4 (both in the pancreatic tissue and plasma) [5] that has anti-inflammatory and anti-diabetic actions [5, 6].

**Fig. 2:**
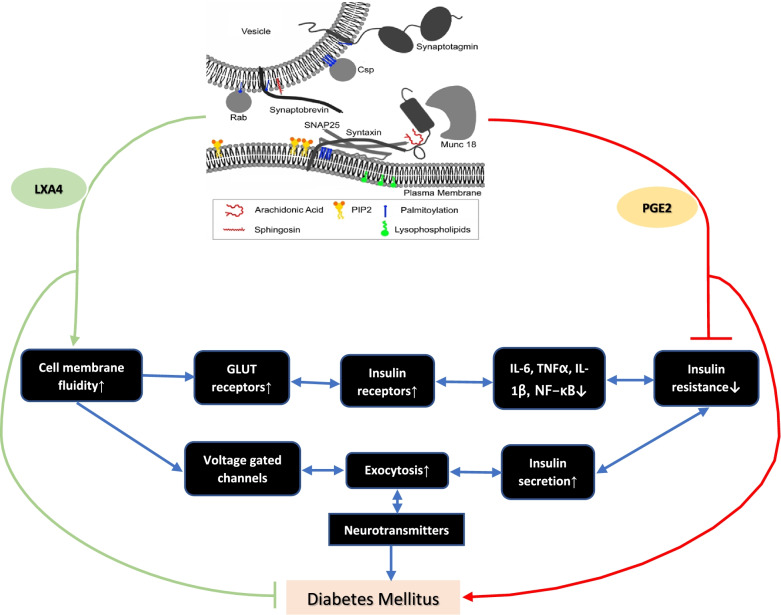
Scheme showing interaction between AA and syntaxin and t he role of LXA4 and PGE2 in the pathobiology of DM. LXA4 is anti-inflammatory whereas PGE2 is pro-inflammatory in nature, and both are derived from AA. For further details see text. DHA, an n-3 fatty acid may also have actions like AA. DHA is the precursor of resolvins, protectins and maresins. LXA4 seems to be the mediator of the anti-inflammatory actions of resolvins, protectins and maresins. LXA4 is more potent compared to resolvins, protectins and maresins in eliciting its anti-inflammatory actions. SNARE proteins = "SNAP REceptor". During membrane fusion, v-SNARE and t-SNARE proteins on separate membranes combine to form a trans-SNARE complex, also known as a "SNAREpin". SNAREs are the core required components of the fusion machinery and function independently of additional cytosolic accessory proteins. When cells containing v-SNAREs contact cells containing t-SNAREs, *trans*-SNARE complexes form, and cell-cell fusion ensues. Munc-18 (an acronym for mammalian uncoordinated-18) proteins are a member of the Sec1/Munc18-like (SM) protein family. Munc-18 proteins are essential components of the synaptic vesicle fusion protein complex and are crucial for the regulated exocytosis of neurons and neuroendocrine cells. PGE2 = Prostaglandin E2; LXA4 = Lipoxin A4. GLUT = Glucose transporter. IL-6 = Interleukin-6; TNF-α = Tumor necrosis factor-α; NF-kB = Nuclear factor-kapa B. PIP2 = Phosphatidylinositol 4,5-bisphosphate or PtdIns(4,5)*P*_2_, also known simply as PIP_2_ or PI(4,5)P_2._

In addition, PUFAs (especially AA) modulate the function of voltage—gated ion channels containing one or several voltage-sensor domains, such as voltage-gated sodium (NaV), potassium (KV), calcium (CaV), and proton (HV) channels, as well as calcium-activated potassium (KCa), and transient receptor potential (TRP) channels—an action by which fatty acids could regulate β-cell membrane potential, and their (β cells) ability to sense plasma glucose and regulate secretion of insulin ([10], see Fig. 2).

### AA can function as a mechanotransducer

Recent studies revealed that pressure and stretch stimuli (including shear stress of blood flow and changes in plasma glucose levels) act as mechanotransducers and produce cellular shape deformation and control their (cell) dynamic behavior including change in their shape, motility, ability to secrete chemicals needed for various physiological actions. Thus, pressure and stretch stimuli regulate phagocytosis (endocytosis and exocytosis), inflammation, immune response, and other functions. These studies suggested that nucleus acts as an elastic mechanotransducer of cellular shape deformation which activates cytosolic phospholipase A2 (cPLA2) that, in turn, results in the release of AA, the precursor of prostaglandins, leukotrienes, thromboxanes, and lipoxins that have potent biological actions including regulation of insulin resistance and insulin secretion [5, 11–15]. Thus, AA functions as a mechanotransducer (pressure and stretch stimuli and glucose activate PLA2 that induces the release of AA from the membrane lipid pool) of various stimuli and influence immune response and development of DM.

### Syntaxin and AA interact with each other to regulate insulins secretion

**Fig. 3:**
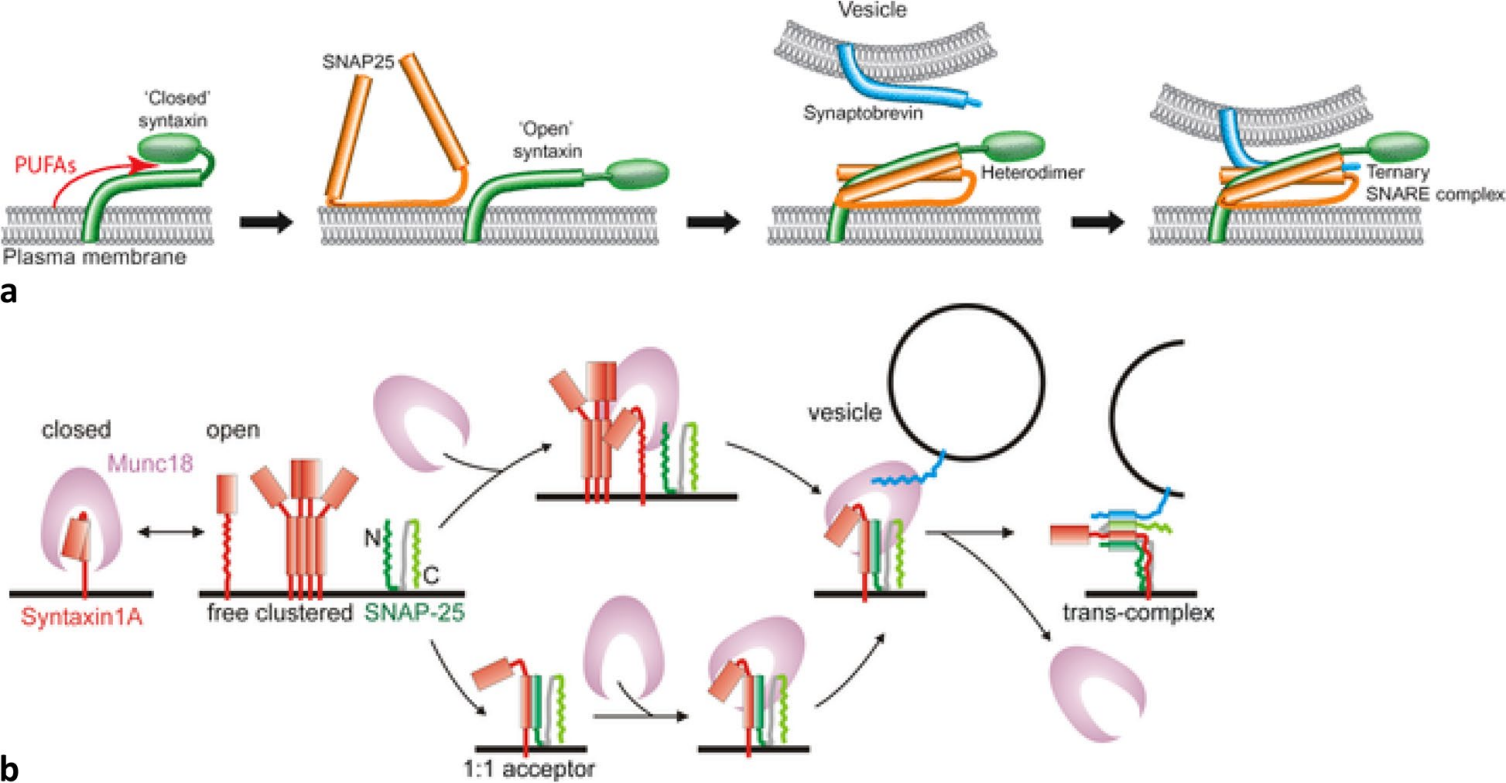
**a** Scheme showing how PUFAs (especially AA and DHA; AA > DHA) interact with syntaxin to regulate exocytosis. PUFAs = Polyunsaturated fatty acids. SNARE complex = The core SNARE complex is a 4-α-helix bundle. Synaptobrevin and syntaxin contribute one α-helix each, while SNAP-25 participates with two α-helices (abbreviated as Sn1 and Sn2). The interacting amino acid residues that zip the SNARE complex can be grouped into layers. Each layer has 4 amino acid residues – one residue per each of the 4α-helices. In the center of the complex is the *zero ionic layer* composed of one arginine (R) and three glutamine (Q) residues, and it is flanked by leucine zippering. Layers '-1', ' + 1' and ' + 2' at the centre of the complex most closely follow ideal leucine-zipper geometry and amino acid composition. **b** Depiction of the formation of a *trans*-SNARE complex. Shows how Munc18 interacts with the SNARE proteins during complex formation

It is noteworthy that syntaxin 4 (STX4), a plasma membrane-localized SNARE protein, has a regulatory role in insulin secretion by human islet β-cells and can preserve β-cell mass by at least, in part, by reducing IFN-γ and TNF-α secretion by inhibiting β cell NF-ĸB expression. In addition, syntaxin 4 enhances CD4^+^ Treg cell function and thus, preserves β cell function [16–18]. AA directly interacts with syntaxin and potentiates exocytosis by interacting with neuronal SNARE complex via Munc18a [19–21]. This interaction between AA and SNARE complex enhances exocytosis of insulin and several neurotransmitters (see Fig. 3). Thus, AA regulates the expression and function of GLUT and insulin receptors (by enhancing the expression of GLUT and insulin receptors) [1–3], in addition to its ability to enhance β cell survival and function. These actions of AA ultimately result in improved insulin secretion and action. Our studies revealed that alloxan-induced type 1 diabetic animals have low plasma, hepatic, and muscle (and possibly, hypothalamic) concentrations of AA in their phospholipid fraction [22, 23]. We also observed that patients with type 2 DM have low plasma concentrations of AA in their phospholipid fraction and LXA4 [24]. These observations imply that AA and LXA4 deficiency predisposes to the development of DM and their (AA and LXA4) administration may be of significant benefit in the prevention of DM [25]. Furthermore, glucose administration reduces hypothalamic AA containing phospholipids (that will be released by the activation of cPLA2 – cytosolic phospholipase A2), that can be metabolized preferentially to prostaglandins, which predominantly have pro-inflammatory actions. These results suggest a critical role for cPLA2—mediated hypothalamic phospholipid AA metabolism in the control of systemic glucose metabolism [26]. Acute administration of glucose to regular chow fed mice activated cPLA2 in the VMH leading to an increase in the production of PGs from neurons that activates VMH (ventromedial hypothalamus) and glucose metabolism in peripheral tissues. In contrast, chronic HFD feeding that also increased cPLA2-mediated PG production from VMH neurons induced hypothalamic inflammation leading to an impairment in peripheral glucose metabolism [26]. In these studies [26], the potential role of LXA4, a potent anti-inflammatory metabolite of AA and PGE2 antagonist was not studied. Thus, the acute effects of PGs (especially PGE2) are different from its chronic actions. Since syntaxin and AA interact with each other and regulate exocytosis (see Figs. 2 and 3), it explains the interwoven relationship among various neurotransmitters, inflammation, and immune response in the pathobiology of DM [22, 27, 28]. It is known that in AA deficiency states there is increased formation of PGE2 that results in inflammation whereas provision of AA in such instances enhanced LXA4 formation with or without any change in PGE2 synthesis [29–32]. This increase in the formation of LXA4 in AA deficiency states in response to AA administration results in a change in the balance between pro- and anti-inflammatory metabolites (PGE2 *vs* LXA4) such that inflammation is suppressed, insulin resistance is decreased that ultimately leads to prevention or abrogation of DM. Thus, systemic administration (oral or intravenous) of AA is expected to reach pancreas and hypothalamus in adequate concentrations and this may result in the prevention and management of DM [22, 27, 28]. It is suggested that methods could be developed to selectively administer AA to pancreas and hypothalamus in liposomal forms of AA.

**Fig. 4:**
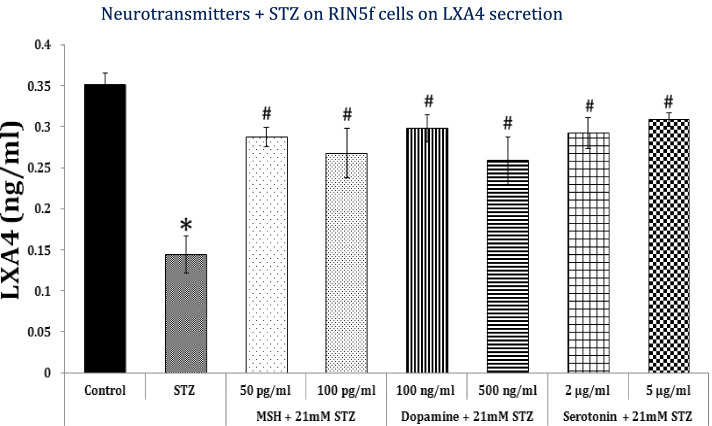
Effect of α-MSH, dopamine and serotonin on STZ-induced suppression of insulin secretion by RIN5F cells. STZ-induced suppression of RIN cells (rat insulinoma) is also abrogated by MSH, dopamine and serotonin and preserved insulin secretory capacity of RIN cells. *P < 0.05 compared to control; #*P* < 0.05 compared to STZ. STZ = Streptozotocin; MSH = Melanocyte stimulating hormone. RIN5F cells = Rat insulinoma cells that secrete insulin

In this context, it is noteworthy that the neurotransmitters α-MSH, dopamine and serotonin not only protected RIN (rat insulinoma) cells against STZ-induced cytotoxicity in vitro but also restored their ability to secrete insulin. Furthermore, α-MSH, dopamine and serotonin (serotonin ≥ dopamine ≥ α-MSH) restored/enhanced LXA4 formation and secretion by RIN cells that was suppressed by STZ (see Fig. 4). These results suggest that an interaction exists between neurotransmitters and AA metabolism which may result in the regulation of insulin secretion and their potential role in the pathophysiology of DM [5, 6, 25, 27].

## Conclusions and future directions

Based on the preceding discussion it is reasonable to propose that AA and its metabolites PGE2 and LXA4, and syntaxin have a critical role in the regulation of glucose homeostasis by virtue of their ability to regulate cell membrane fluidity, expression of GLUT and insulin receptors, insulin exocytosis, and inflammation (especially AA, PGE2 and LXA4 by their negative feedback control on the secretion of IL-6, TNF-α and NF-kB expression) [1, 2, 4, 5, 13–23, 28, 31–38]. In addition, AA and other unsaturated fatty acids regulate the function of various voltage gated ion channels that are critical to insulin secretion ([6], see Fig. 2). Syntaxin, which is needed for pancreatic β cell survival and secretion of insulin, is regulated by AA [19–21, 37, 38]. Furthermore, AA regulates insulin exocytosis from β cells by interacting with syntaxin and regulating its expression [13–21]. AA regulates the exocytosis of several neurotransmitters that may explain the close interaction and the regulatory role of various neurotransmitters in insulin secretion, action and in the pathobiology of DM (especially type 2 DM) [33–39]. Thus, it is likely that high fat diet induces hypothalamic inflammation by augmenting the production of PGE2 from AA that led to abnormalities in the release and action of neurotransmitters acetylcholine, serotonin, GABA (gamma-aminobutyric acid), dopamine and α-MSH (alpha-melanocyte stimulating hormone) [26, 27, 39]. It is known that cholesterol and saturated fatty acids inhibit formation of AA from its dietary precursor linoleic acid (LA) and augment the formation of PGE2 that may explain as to why high fat diet causes hypothalamic inflammation [1, 31, 32]. On the other hand, availability of adequate amounts of AA enhances the formation of LXA4, a potent anti-inflammatory compound, that has antidiabetic actions [5, 6, 25, 27, 28, 39, 40]. Thus, it is suggested that availability of AA prevents both high fat diet and chemical-induced DM by enhancing LXA4 formation and prevents or even reverses hypothalamic inflammation and dysfunction in addition to its ability to regulate syntaxin. Our recent studies showed that several neurotransmitters protect pancreatic β cells from the cytotoxic action of STZ and enhance LXA4 formation and release, implying a close interaction between neurotransmitters and AA and LXA4 in the pathobiology of DM (see Fig. 4). Based on these studies it is proposed that oral or parenteral administration of AA and LXA4 may form a new approach to prevent DM.

In this context, it is noteworthy that DHA content of brain is high (DHA > AA > EPA) and forms the precursor of potent anti-inflammatory compounds resolvins, protectins and maresins. But the cell membrane content of DHA is less compared to AA. DHA increases cell membrane fluidity (DHA > AA) in view of its highly unsaturated nature (contains 6 double bonds and 22 carbons) and participates in exocytosis especially of neurotransmitters and insulin [13–15, 33–38]. In our studies, it was noted that AA is more potent than DHA in preventing chemical and high fat diet-induced DM [5, 22–25, 27, 28, 40], implying that perhaps a combination of AA and DHA is worthwhile in the prevention and management of DM. One of the functions of AA and DHA in the brain is to regulate exocytosis of various neurotransmitters, inhibit hypothalamic inflammation, suppress release of excess IL-6 and TNF-α to protect neuronal cells from various endogenous and exogenous injuries and thus, regulate neuronal integrity and function.

In addition, AA and DHA form an important constituent of all cell membranes and thus, regulate cell membrane fluidity and the expression of several types of receptors including but not limited to GLUT, and insulin, and their affinity to their corresponding proteins/hormones/growth factors. AA and DHA interact with syntaxin to regulate exocytosis of insulin and neurotransmitters in the brain, possess anti-inflammatory actions by themselves and by virtue of their ability to form precursors to anti-inflammatory LXA4 (from AA), and resolvins, protectins and maresins (from DHA) and show cytoprotective actions. By virtue of these actions AA and DHA are amicably suited to prevent DM induced by various exogenous and endogenous agents. This implies that AA and DHA and their anti-inflammatory metabolites (LXA4, resolvins, protectins and maresins) function as endogenous anti-diabetic molecules. This proposal is supported by our previous studies which showed that AA, DHA, LXA4, resolvins and protectins prevent DM in experimental animals [4–6, 16, 17, 22, 23, 25, 27, 41–44]. The anti-diabetic actions of AA and DHA are predominantly due to the increased formation of LXA4, resolvins, protectins and maresins though possibility that AA and DHA themselves have antidiabetic actions cannot be discounted. Hence, it may be argued that any interference with the conversion of AA and DHA to their respective anti-inflammatory metabolites may not result in the prevention of development of DM. Furthermore, the administered AA and DHA need to reach pancreatic $\beta \mathrm{cells}$ in adequate amounts such that they could be converted to their respective anti-inflammatory metabolites to prevent DM. Hence, understanding the molecular mechanisms involved in the conversion of AA and DHA to their respective anti-inflammatory metabolites is of paramount importance. Another attractive proposal is to administer LXA4/resolvins/protectins/maresins themselves to prevent DM. LXA4/resolvins/protectins/maresins have very short half-life (few seconds to few minutes) and are not active when administered orally. Hence, methods need to be developed such that they can be delivered direct to pancreatic β cells. It is likely that administration of AA/DHA throughout life starting from fetal life could be one strategy that may render the availability to these fatty acids in adequate amounts to β cells such that DM prevention is possible [45–48]. This is supported by the observation that infants who were breast fed (breast milk is rich in AA and DHA) and received perinatal supplementation of PUFAs have lower incidence of DM [49–51]. In such an instance, it may be prudent to measure plasma levels of LXA4, resolvins, protectins and maresins to know whether there is indeed increased formation of these anti-diabetic molecules because of administration of their precursors. These measurements may be done in addition to evaluating the plasma levels of insulin, C-peptide, adiponectin, IL-6, TNF-α, insulin resistance indices and neurotransmitters serotonin, dopamine, catecholamines, α-MSH, etc., and syntaxin in the peripheral circulating leukocytes and lymphocytes [52–56] (that have the complete systems for all neurotransmitters and so may reflect the events in the hypothalamus) to know whether the administered AA/DHA could reach hypothalamus to regulate β cell function. Thus, a comprehensive evaluation of potential anti-diabetic role of AA/DHA needs to be performed in future in humans.

## Data Availability

All data and materials are given in the manuscript.
